# Supplementary material for: Preparation and Characterization of Polyvinylpyrrolidone/Cellulose Nanocrystals Composites
Source: Nanomaterials (Basel). 2018 Dec 5;8(12):1011. doi: 10.3390/nano8121011 (PMC6315985; doi:10.3390/nano8121011)
Supplement: Supplementary file 1 [file nanomaterials-08-01011-s001.pdf]

Supplementary Material

Preparation and Characterization of Polyvinylpyrrolidone/Cellulose Nanocrystals Composites

Marina Voronova [1], Natalia Rubleva [1], Nataliya Kochkina [1], Andrei Afineevskii [2], Anatoly Zakharov [1] and Oleg Surov [1,*]

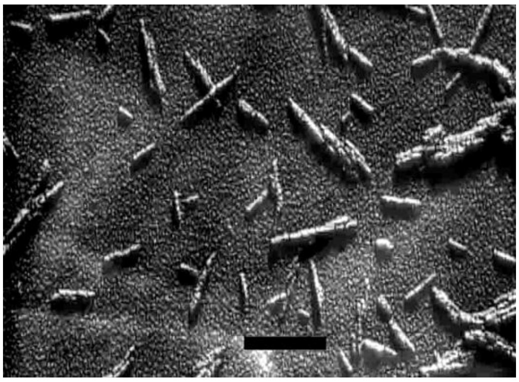

Figure S1. TEM image of CNC. The scale bar is 100 nm.

Table S1. CNC characteristics.

Parameter	CNC characteristics
[1] Dimensions, nm	
length	100–150
diameter	15–20
[2] Hydrodynamic diameter, nm	100
[3] Total sulfur content, %	0.6
[4] Degree of polymerization	80
[5] Crystallinity index, %	84.0
[5] Crystalline dimension by (200) plane, nm	4.0

[1] TEM. [2] DLS. [3] Elemental analysis. [4] In terms of viscosity of CNC solution in cadoxene. [5] X-ray diffractional analysis.

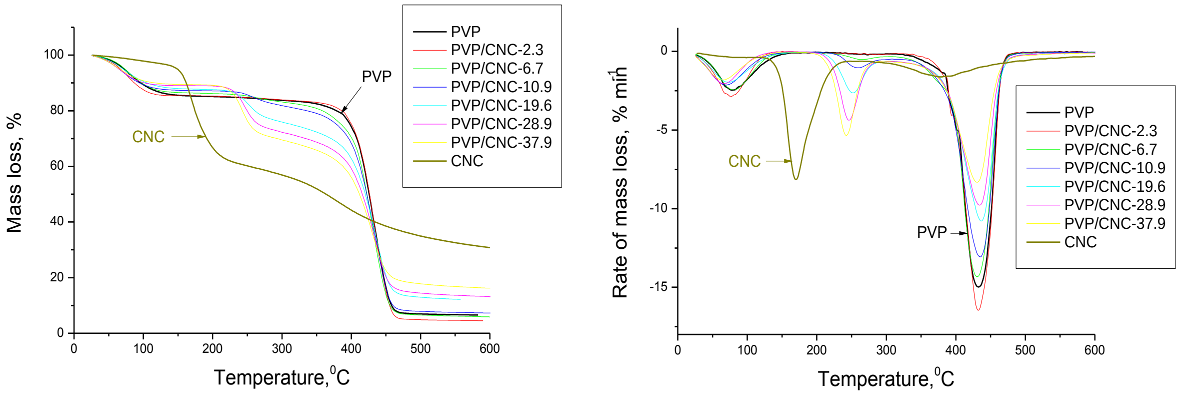

(**a**) (**b**)

Figure S2. TG (**a**) and DTG (**b**) curves of the PVP/CNC composite films.

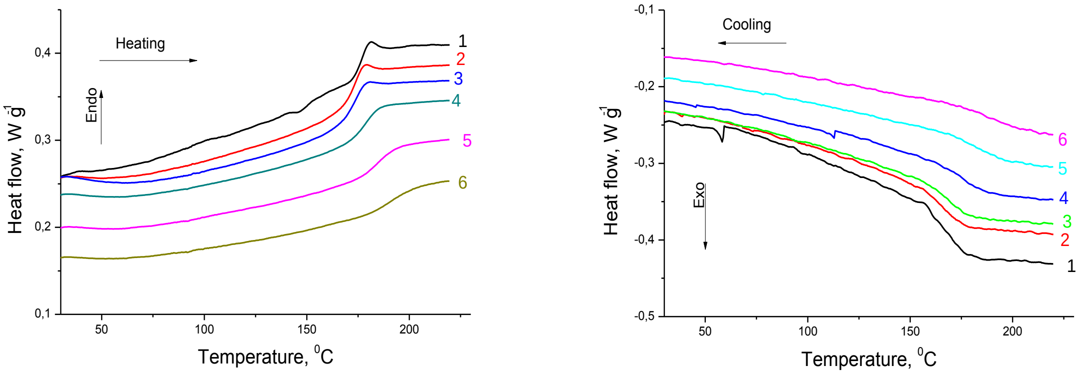

(**a**) (**b**)

Figure S3. DSC traces for heating (**a**) and cooling (**b**) of the PVP/CNC composite films: 1 - neat PVP; 2 - PVP/CNC-4.6; 3 - PVP/CNC-10.9; 4 - PVP/CNC-19.6; 5 - PVP/CNC-28.9; 6 - PVP/CNC-37.9.

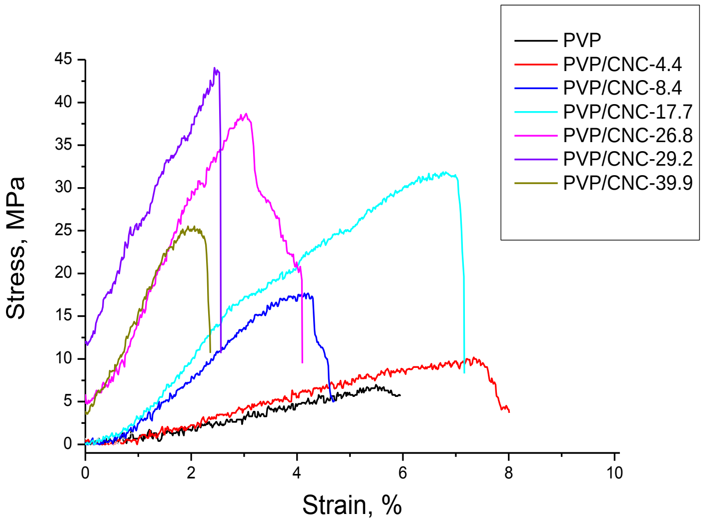

Figure S4. Typical stress-strain curves of the PVP/CNC composite films.

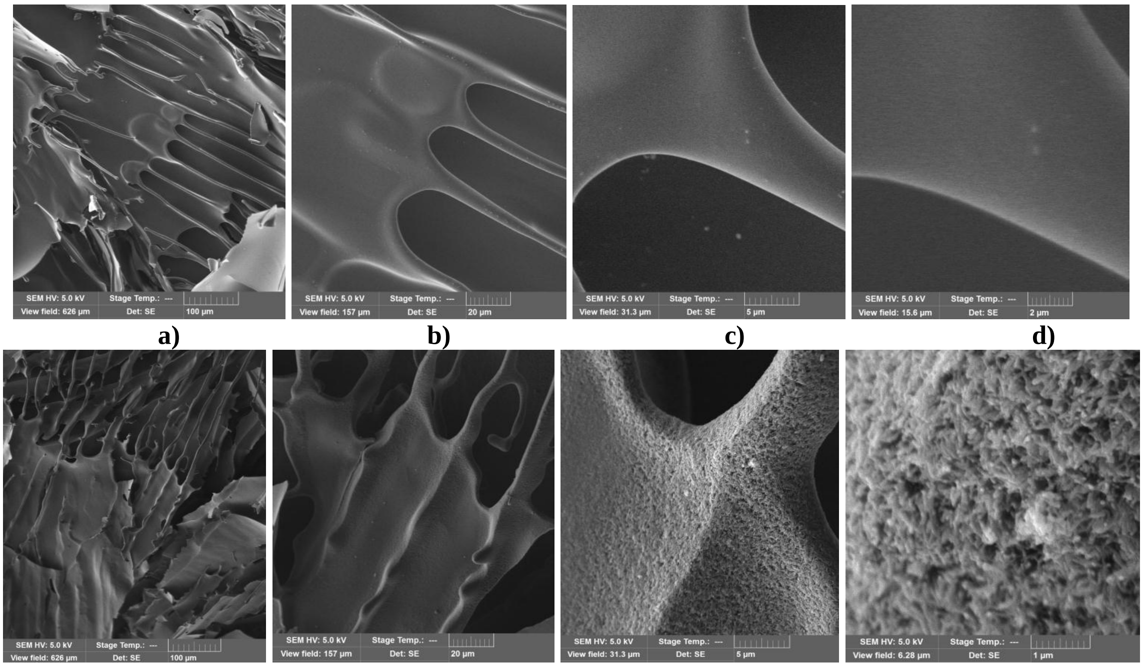

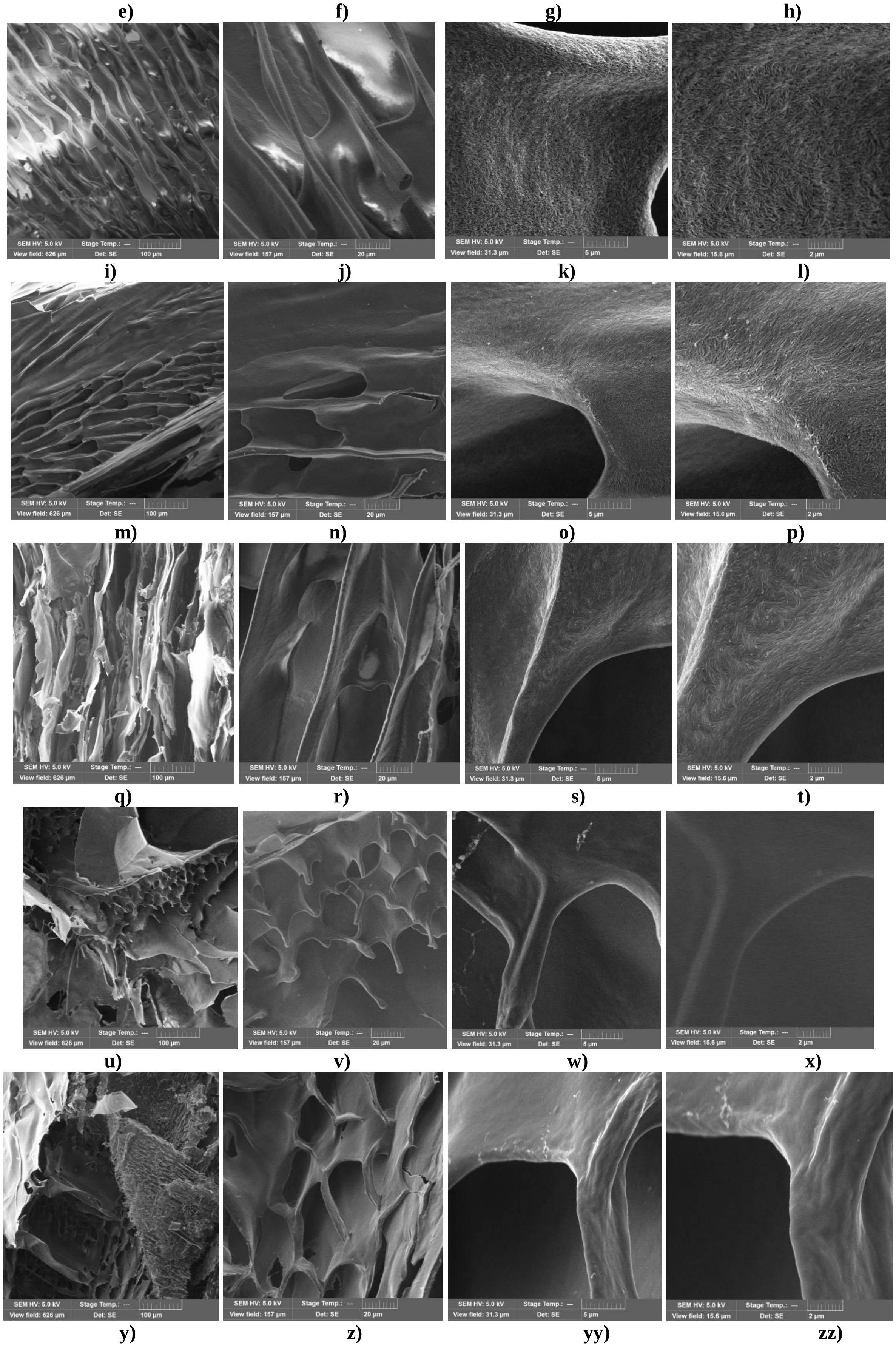

Figure S5. SEM images of the neat PVP aerogel (**a-d**) and the PVP/CNC composite aerogels with CNC content (wt.%) of: 4.6 (**e-h**); 10.9 (**i-l**); 19.6 (**m-p**); 28.9 (**q-t**), 54.5 (**u-x**); 70.6 (**y-zz**), respectively. Scales: 100 µm (**a, e, i, m, q, u, y**); 20 µm (**b, f, j, n, r, v, z**); 5 µm (**c, g, k, o, s, w, yy**); 2 µm (**d, l, p, t, x, zz**); 1 µm (**h**).

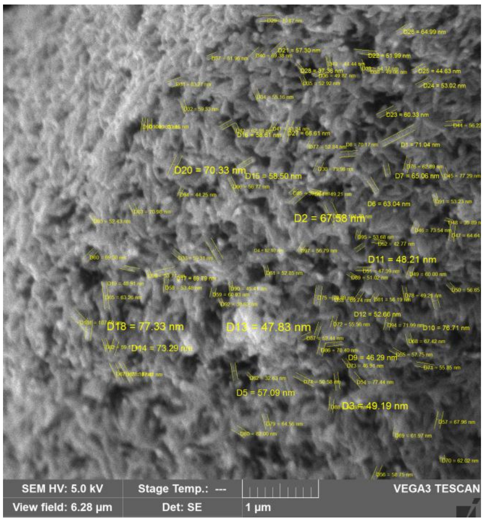

(**a**)

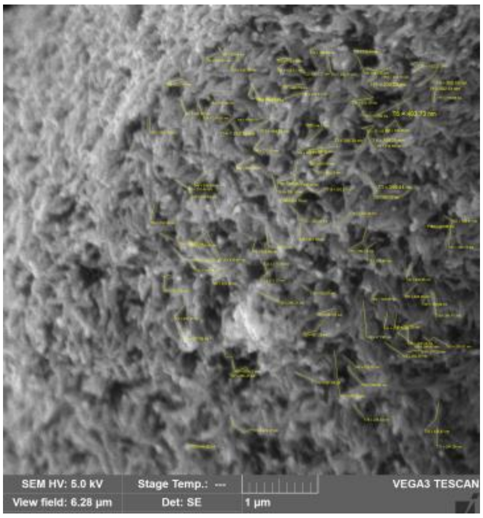

(**b**)

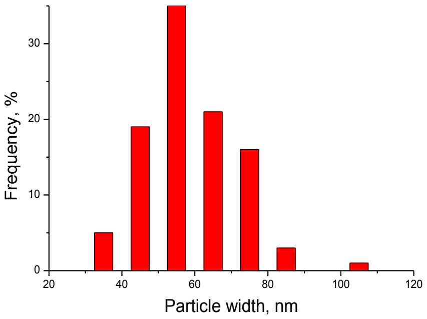

(**c**)

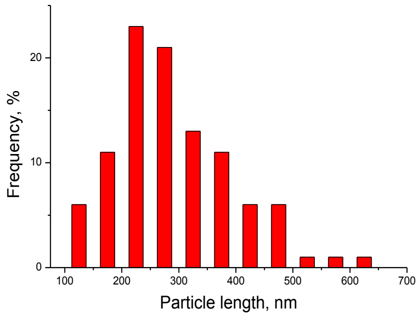

(**d**)

Figure S6. SEM images of the PVP/CNC-4.6 aero for CNC particle size estimation across the widths (**a**) and along the lengths (**b**). The scale bar is 1 μm. The CNC particle size distribution across the widths (**c**) and along the lengths (**d**).

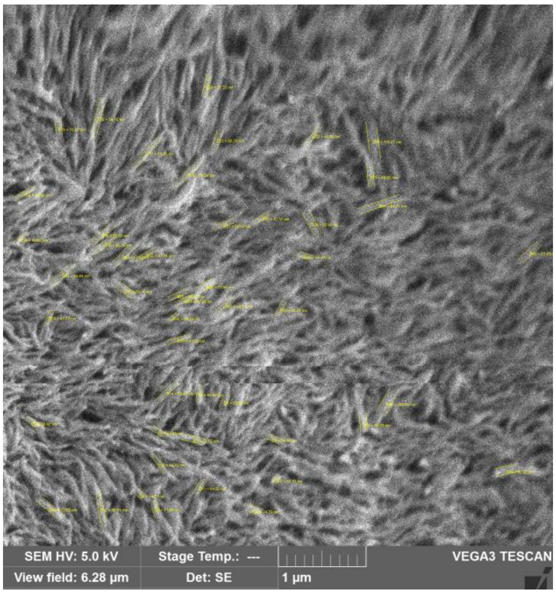

(**a**)

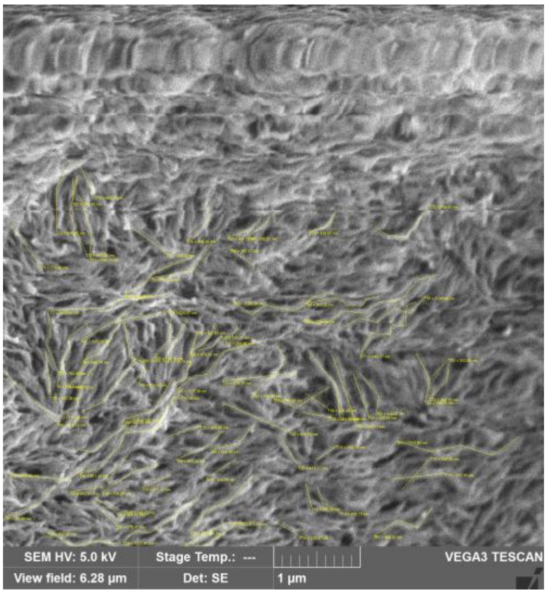

(**b**)

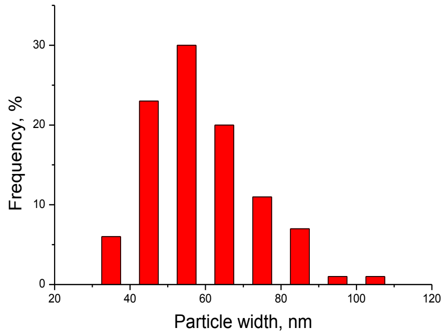

(**c**)

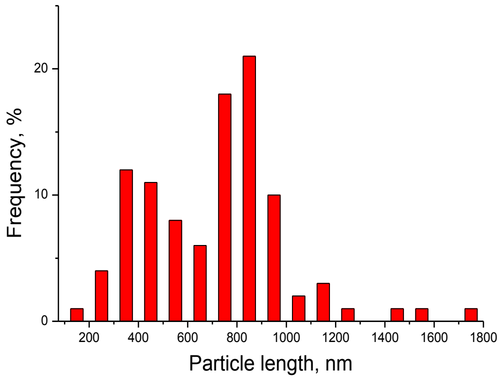

(**d**)

Figure S7. SEM images of the PVP/CNC-10.9 aero for CNC particle size estimation across the widths (**a**) and along the lengths (**b**).The scale bar is 1 μm. The CNC particle size distribution across the widths (**c**) and along the lengths (**d**).

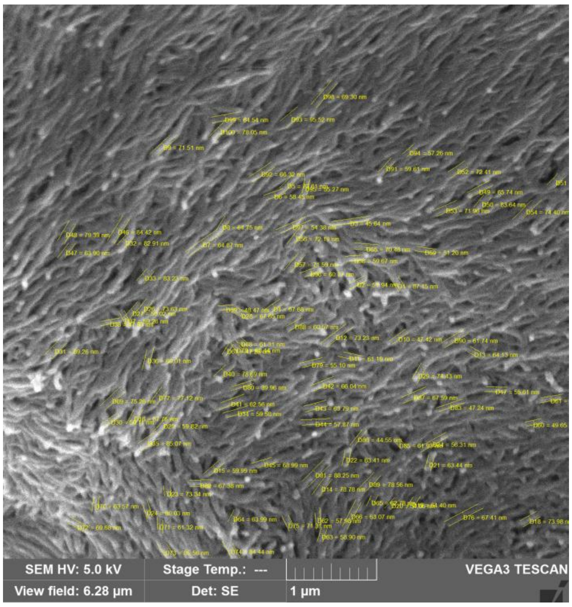

(**a**)

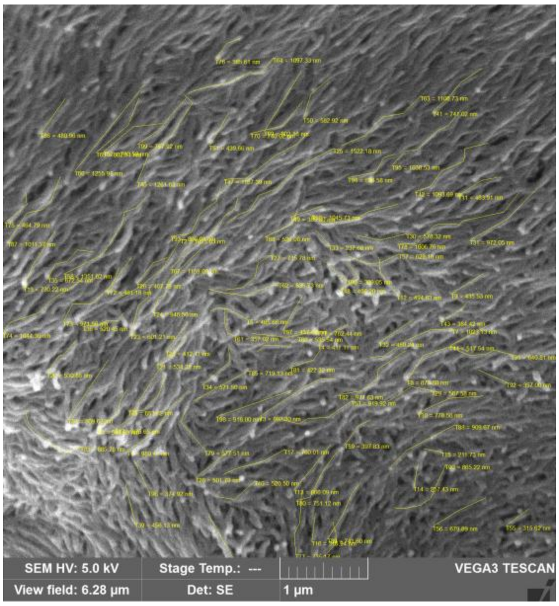

(**b**)

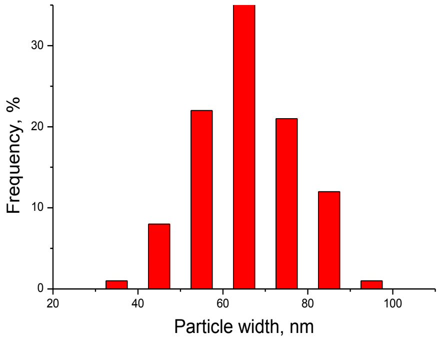

(**c**)

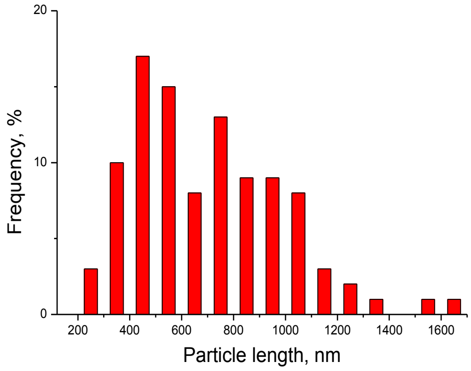

(**d**)

Figure S8. SEM images of the PVP/CNC-19.6 aero for CNC particle size estimation across the widths (**a**) and along the lengths (**b**). The scale bar is 1 μm. The CNC particle size distribution across the widths (**c**) and along the lengths (**d**).

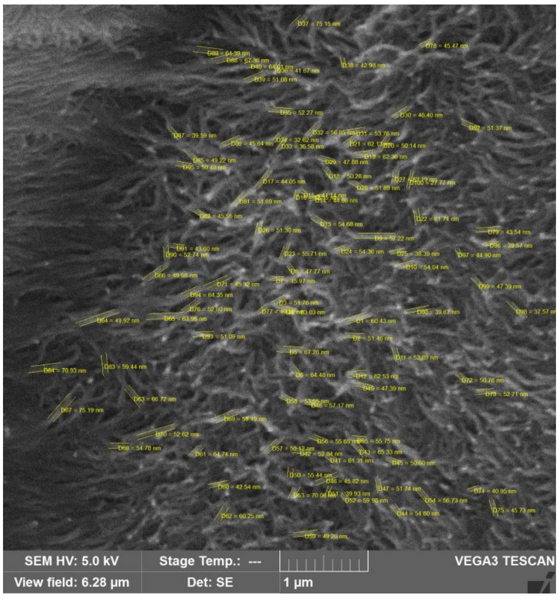

(**a**)

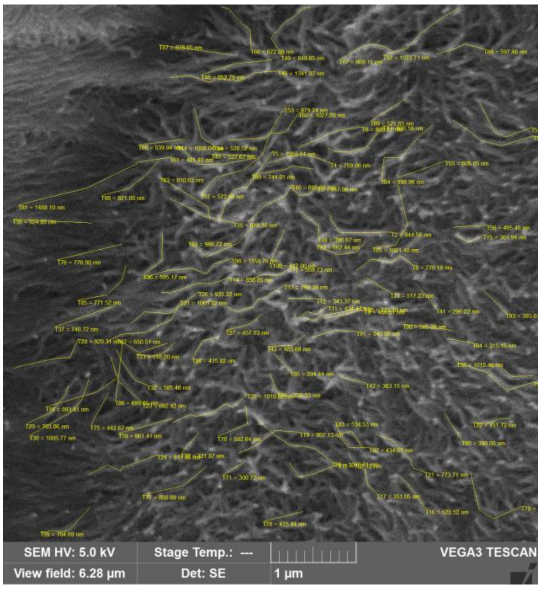

(**b**)

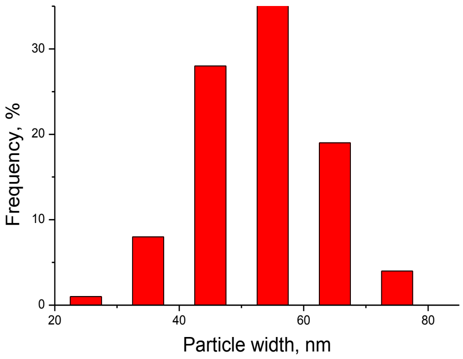

(**c**)

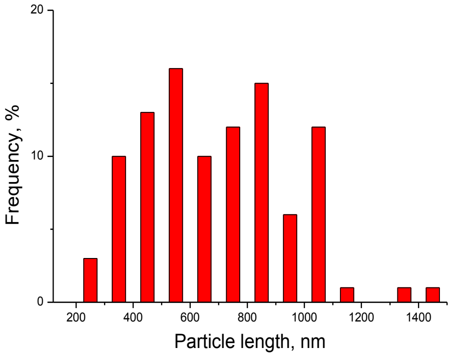

(**d**)

Figure S9. SEM images of the PVP/CNC-28.9 aero for CNC particle size estimation across the widths (**a**) and along the lengths (**b**). The scale bar is 1 μm. The CNC particle size distribution across the widths (**c**) and along the lengths (**d**).

Water

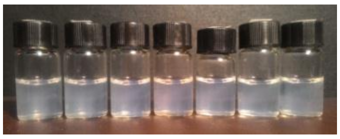

Immediately after redispersion

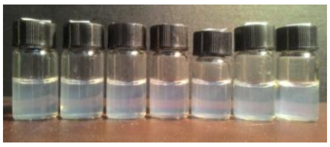

After 10 days

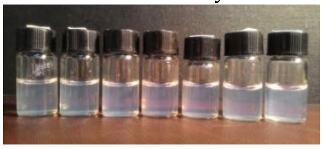

After a month

Propanol

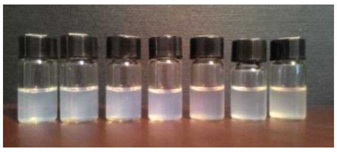

Immediately after redispersion

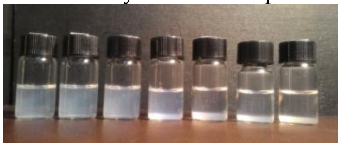

After 3 days

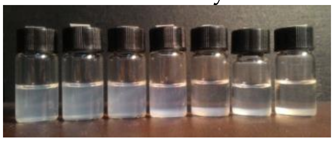

After 10 days

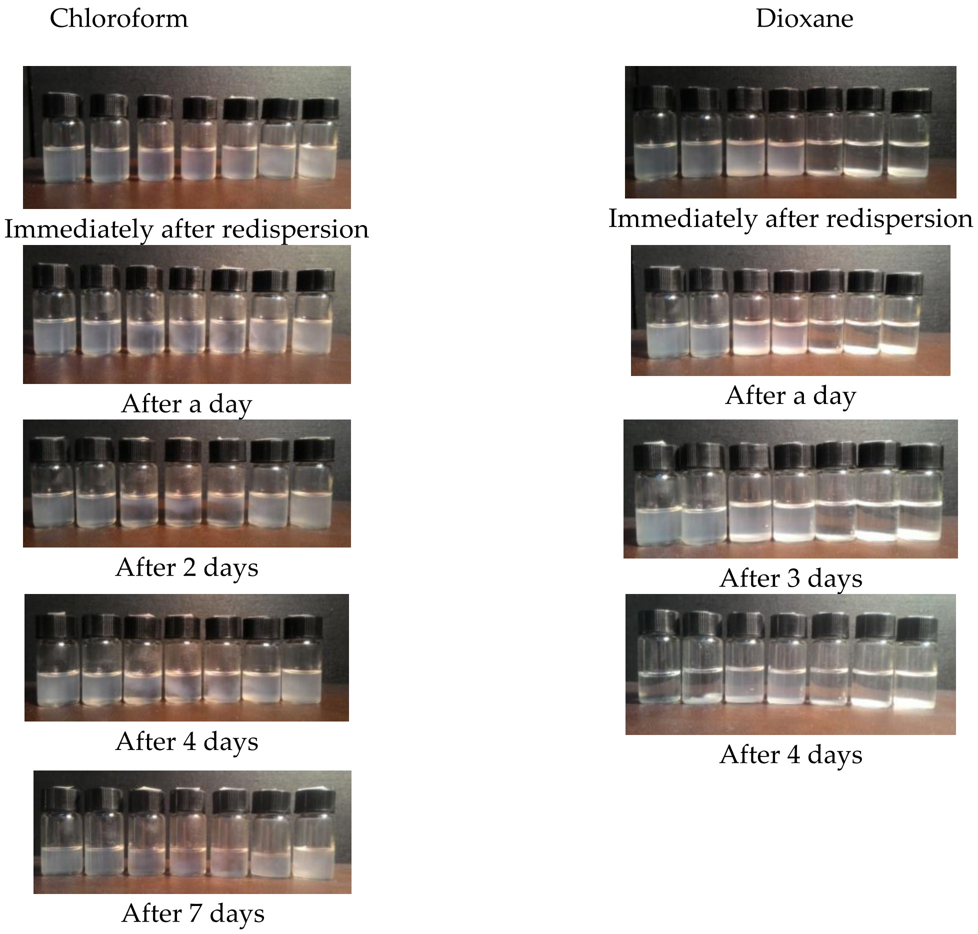

Figure S10. Photos of the redispersed suspensions during storage. From the left to the right: PVP/CNC-4.6 aero; PVP/CNC-10.9 aero; PVP/CNC-16.3 aero; PVP/CNC-19.6 aero; PVP/CNC-28.9 aero; PVP/CNC-37.9 aero; PVP/CNC-54.5 aero. The CNC concentration in the suspensions is 0.2 wt.%.

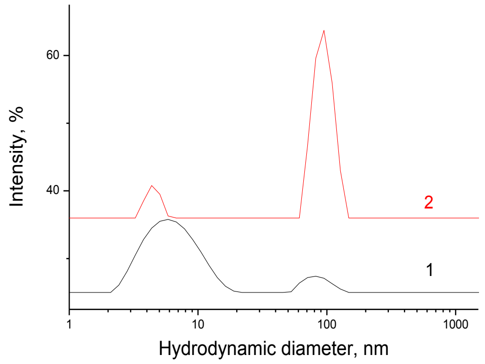

Figure S11. PVP particles size distribution in propanol (1) and chloroform (2) (the solution concentration is 0.2 wt.%).
